# Stakeholders' perspectives on HIV prevention in the Czech Republic: a qualitative study

**DOI:** 10.3389/fpubh.2026.1764708

**Published:** 2026-05-29

**Authors:** Veronika Szépe, Sylva Rödlová, Milan Zlámal, Aleš Tichopád

**Affiliations:** 1 Department of Biomedical Technology, Faculty of Biomedical Engineering, Czech Technical University in Prague, Prague, Czechia; 2 Department of Hygiene, Third Faculty of Medicine, Charles University, Prague, Czechia; 3 Clinic of Infectious Diseases, First Faculty of Medicine, Charles University and Military University Hospital, Central Military Hospital – Military University Hospital, Prague, Czechia

**Keywords:** Czech Republic, HIV prevention, PrEP, stakeholders, testing

## Abstract

**Introduction:**

The present study explores stakeholder perspectives on HIV prevention in the Czech Republic, with a focus on perceived gaps and priorities in current strategies.

**Methods:**

Semi-structured interviews were conducted with 11 institutional stakeholders, including representatives of governmental institutions, non-governmental organizations, healthcare providers, insurers, and the pharmaceutical sector. The present study combined qualitative content analysis with quantitative analysis of coded data.

**Results:**

It was determined by stakeholders that men who have sex with men (MSM) represent the primary focus of current HIV prevention efforts. Concurrently, certain stakeholders emphasized the necessity to reinforce preventive measures for additional demographic groups, most notably migrants and heterosexual populations. Transgender individuals and prisoners were mentioned less frequently, indicating their limited visibility in stakeholder discussions. The primary challenges identified included funding constraints, a lack of reimbursement for PrEP, stigma, and inadequate coordination of prevention strategies. These findings are indicative of stakeholder perspectives and should be interpreted as exploratory.

**Discussion:**

The report identifies areas for further research and potential discussion, particularly regarding the balance of prevention efforts across different population groups and the need for improved coordination and access to prevention tools.

## 1 Introduction

Significant advancements have been made in the field of HIV prevention across Europe over the past decade. However, notable regional disparities persist, particularly in Central and Eastern Europe. In the Czech Republic, relatively low HIV prevalence (4,100 people living with HIV in 2023) conceals rising incidence rates (200 new HIV infections in 2023) (1), with increasing challenges among key populations such as men who have sex with men (MSM), migrants and people who inject drugs (PWID) (2).

The most efficacious HIV prevention strategies are those that are tailored to the specific needs of the at-risk population. In the Czech Republic, the principal high-risk groups are MSM, PWID, sex workers, and migrants. Each group is confronted with distinctive challenges that impact their vulnerability and access to prevention services. MSM represent the largest proportion of new HIV diagnoses in the Czech Republic. Stigma, fear of discrimination and social taboos concerning homosexuality frequently impede the ability of MSM to seek testing and prevention services. To address these barriers, it is necessary to implement targeted outreach, peer-led interventions, and in- risk community-based testing programs (3). PWID are at considerable risk of HIV infection due to the practice of needle sharing and the lack of access to clean syringes. The implementation of harm reduction strategies, including needle exchange programs and opioid substitution therapy, has been demonstrated to be an effective approach in reducing the transmission of HIV among this group. However, there remains inconsistency in the implementation of these strategies across the country (4). The third group comprises individuals engaged in sexual activities in exchange for remuneration. Despite the paucity of data on HIV prevalence among sex workers in the Czech Republic, international studies suggest that this group is at an elevated risk due to unsafe sexual practices and barriers to healthcare access related to lifestyle and stigma. The implementation of condom distribution and regular testing programs represents a crucial intervention strategy for this population (2). Migrants, in particular those originating from regions with elevated HIV prevalence, confront a multitude of obstacles, including linguistic barriers, cultural dissimilarities, and disrupted access to healthcare. The influx of Ukrainian refugees in 2022 has brought into sharp focus the necessity for the implementation of culturally sensitive and accessible prevention strategies (5).

The prevention of HIV in the Czech Republic can be categorized into three principal types: primary, secondary, and tertiary prevention. The objective of primary prevention is to halt the incidence of new infections by providing education, distributing condoms, and implementing harm reduction programs (6). Secondary prevention entails the early detection of HIV through the implementation of widespread testing and the immediate linkage of individuals to care. Tertiary prevention is concerned with reducing the complications associated with HIV infection through the provision of effective treatment and care, based on the Undetectable = Untransmittable (U = U) principle (7).

The advent of pre-exposure prophylaxis (PrEP) and antiretroviral therapy (ART) represents a significant advancement in the field of biomedical HIV prevention on a global scale. PrEP has been demonstrated to be an efficacious intervention for the reduction of new infections among high-risk populations, including MSM, sex workers, and serodiscordant couples. France and the United Kingdom have achieved notable reductions in HIV incidence through the widespread uptake of PrEP and the implementation of accessible public health frameworks (8). In the Czech Republic, however, the availability and uptake of PrEP remain limited. Despite the reduction in the cost of generic PrEP, the lack of public reimbursement schemes and insufficient awareness among healthcare providers and potential users represent significant barriers to its uptake (6). In contrast, the Czech Republic has achieved high coverage (95.5%) of ART in 2024 (9), achieved the UNAIDS target of 95% of people living with HIV. 97.5% of people diagnosed with HIV who undergo treatment achieve viral suppression (9), which again meets the UNAIDS target of 95% of HIV-positive people receiving treatment (10). This is consistent with the “Undetectable = Untransmittable” (U = U) principle, a global campaign ruled by Centre for Disease Control and Prevention (CDC) that emphasizes that individuals on effective ART are unable to sexually transmit the virus (11).

The effectiveness of HIV prevention in the Czech Republic depends on the cooperation and contributions of many stakeholders, including governmental institutions, healthcare providers, non-governmental organizations (NGOs), and the private sector, such as pharmaceutical companies, industrial enterprises, etc. Each stakeholder group fulfills a specific role in efforts to overcome structural, social, financial, and individual barriers to prevention. The aim of this study is to explore stakeholder perspectives on HIV prevention in the Czech Republic using qualitative interviews and content analysis, with a focus on perceived priorities, barriers, and opportunities.

## 2 Materials and methods

The literature reveals a diversity of methodologies employed in stakeholder analysis. Predominantly, stakeholder analyses incorporate both normative and instrumental approaches across various disciplines and contexts, utilizing a comprehensive array of methods. The procedural framework for stakeholder analysis typically unfolds in the following stages: (1) identification of stakeholders; (2) differentiation and categorization of stakeholders; (3) reviewing the relationships between stakeholders and examining attitudes and opinions on this topic.

### 2.1 The timing and scope

This was qualitative research conducted in the period from 1 November 2024 to 19 February 2025. The semi-structured qualitative interviews were conducted with open questions among stakeholders with a professional or otherwise relevant relationship to the issue of HIV prevention in Czech Republic. The questioning focused in detail on the concept of the HIV prevention and its feasibility, barriers, benefits, and opportunities for the Czech Republic.

### 2.2 Identification of stakeholders

We employed targeted snowball (chain-referral) sampling (12) to identify stakeholders for participation in our research, as no comprehensive a stable sampling frame of relevant actors in HIV prevention exists and stakeholders are complemented in professional and institutional networks. All potential participants received a uniform email invitation for engagement. Initial participants were selected from major institutions involved in HIV prevention and subsequently referred additional relevant stakeholders, with recruitment proceeding iteratively through this referral chain. Selection criteria for stakeholders were predicated upon their rank and role, giving priority to senior representatives who had had a direct historical engagement in HIV prevention. We contacted 16 representatives of stakeholders involved in HIV prevention in the Czech Republic, 11 of whom responded positively and participated in the interviews. All stakeholder categories identified within our sampling frame were represented in this study. As a non-probability method, snowball sampling may preferentially include more visible or well-connected actors and underrepresent less connected stakeholders; therefore, the sample reflects perspectives within the identified professional network rather than a statistically representative set of all stakeholders.

### 2.3 Differentiation and categorization of stakeholders

For our research, we decided on a stakeholder categorization approach inspired by Lübbeke (13) which identifies the main stakeholders in healthcare as patients, providers (professionals and institutions), payers and policymakers (often referred to as “the four Ps” in healthcare), as well as industry, regulators, research community, and media. In the first phase, a map of identified stakeholders was generated and subsequently divided into five occupational groups as a slight modification of the framework, reflecting the specifics of the HIV prevention issue. This categorization was based on recognizing that each group holds unique perspectives and interests that are crucial for understanding and evaluating the possibilities and challenges associated with HIV prevention in the Czech Republic. This categorization was used to ensure that perspectives across key stakeholder domains were captured and analytically structured. These groups are: Representatives of the MoH (denoted as MoH cohort), Representatives of insurance companies (IC), Representatives of pharma and their two local associations (Ph), Non-government organizations (NGO) and Healthcare professionals (HP). We did not directly involve patients or people from the risk community in this study. We invited representatives of a patient organization, which is included in the NGO group in this study.

### 2.4 Exploration of attitudes and opinions on the topic

A semi-structured interview based on a predefined scenario covering key areas of the subject discussed was applied. The key areas were: Key population, Education, Expectations related to HIV prevention, Barriers, and challenges related to the HIV prevention, Funding, and resources to HIV prevention. The interviews were conducted and recorded online in the Microsoft Teams platform or face-to-face interviews and then transcribed, ensuring that even colloquialisms, jargon and vernacular expressions were captured accurately. All interviews were anonymized.

### 2.5 Content analysis

Two researchers independently coded all the data. The content analysis principles were applied (14). The units of analysis were defined at the level of phrases or sentences that conveyed a complete idea or concept. Each unit was categorized based on aspects central to HIV prevention, such as stakeholder expectations, barriers and challenges, funding, and resources. The researchers systematically reviewed the textual data. Each relevant segment of text was assigned a code corresponding to the predefined cluster. Each code associated with the cluster in which it is located may be mentioned several times in a single transcript. This process was iterative, with the research team regularly discussing and resolving any discrepancies in coding to ensure reliability and consensus. Once coding was completed, the coded data were analyzed to identify common patterns, themes or emerging trends related to HIV prevention. The coding and analysis of codes were conducted via MAXQDA Analytic Pro (24.7.0) software. In addition to qualitative content analysis, quantitative analysis of codes data was performed to examine the distribution of responses across stakeholder groups. The frequency of coded responses within each thematic cluster was analyzed using Fisher's exact test and, where appropriate, Chi-square test, depending on data distribution and cell counts. These tests were selected due to the categorical nature of data and small sample size. Given the exploratory design and limited number of participants, statistical results are interpreted descriptively and in conjunction with qualitative findings rather than as confirmatory inferential evidence.

## 3 Results

A total of 11 prominent stakeholders were approached for participation, all with demonstrable expertise in the subject matter. They were segmented into five distinct cohorts based on their affinity with the subject matter, each cohort encompassing 1–3 respondents (Table 1). Upon review by two independent researchers, a coding paradigm was obtained comprising five top clusters termed Key population, Education, Expectations, Barriers and Challenges, Funding, and Resources. Each comprising a variable number of codes obtained from the transcript analysis.

**Table 1:** Number of participating stakeholders by groups.

Groups (*N* = 5)	# of stakeholders (*N* = 11)
MoH	3
NGO	3
IC	2
HP	2
Ph	1

### 3.1 Key population

**Figure 1:**
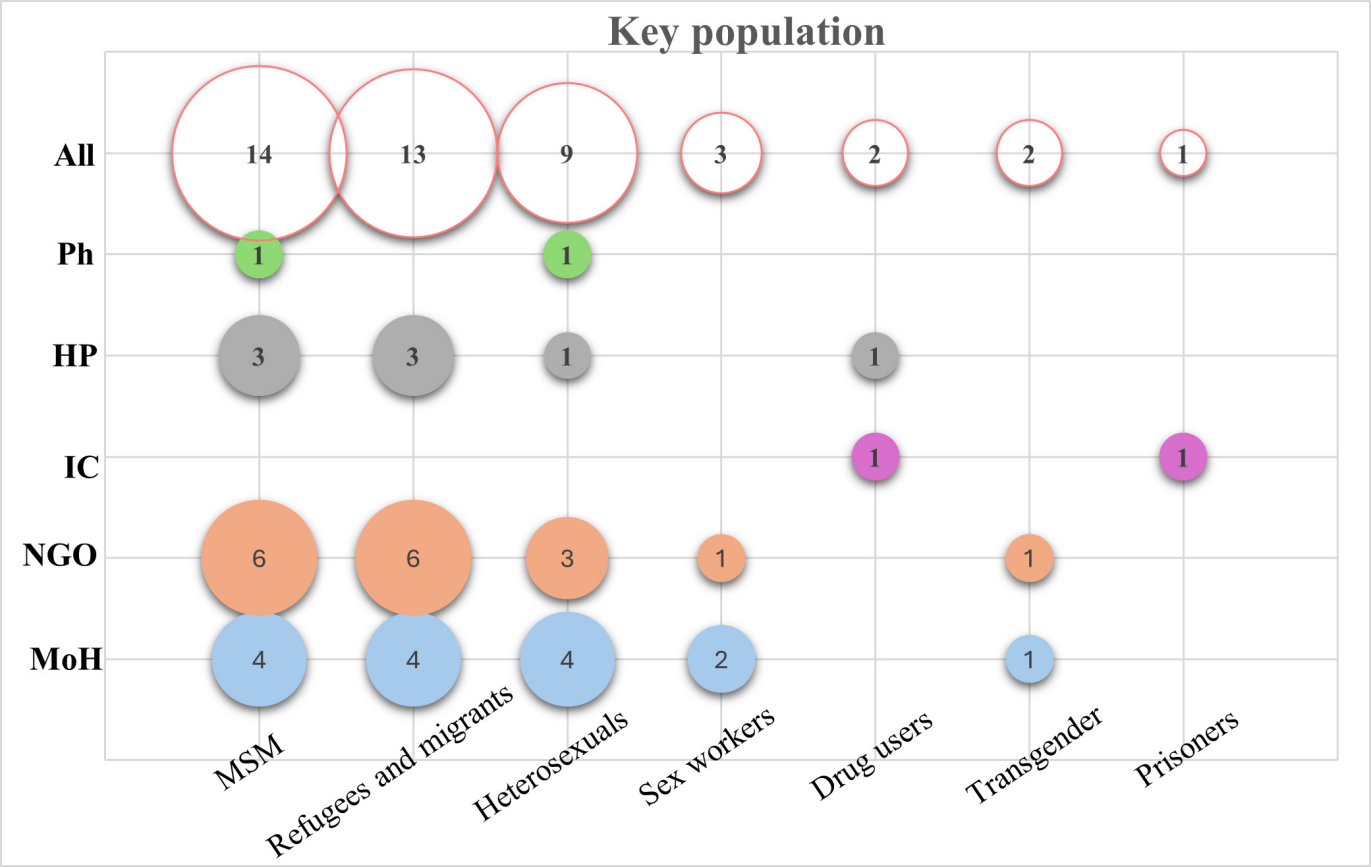
“Key population” cluster derived from qualitative content analysis of stakeholder interviews. The figure displays the distribution of coded mentions of specific population groups (e.g., MSM, migrants, drug users) across stakeholder groups involved in HIV prevention in the Czech Republic. Bubble size represents the frequency with which each population group was mentioned within the coded data, indicating its relative prioritization in prevention efforts. Larger bubbles reflect greater emphasis placed on a given population by stakeholders.

Within the key population cluster, we examined which groups in the general population have the greatest need and potential for HIV prevention. Figure 1 illustrates how key populations are prioritized in HIV prevention efforts across various stakeholders in the Czech Republic. The “Key population” cluster reveals the part of the population that is most affected by HIV prevalence or that needs the most attention in HIV prevention. This cluster includes seven codes: MSM (men who have sex with men), drug users, refugees from Ukraine, heterosexuals, sex workers, transgender, prisoners (Figure 1).

Based on coded stakeholder responses, MSM were most frequently mentioned, particularly from NGO and the MoH groups, making them overrepresented in prevention strategies (*p* < 0.05). Refugees and migrants, who partially overlap with the heterosexual category, also receive notable attention but not as consistently across all stakeholder groups. Heterosexuals appear in multiple key population groups, partly due to their inclusion within the refugee and migrant population, which complicates their representation. Sex workers are moderately represented in prevention efforts, but their inclusion is not as strong as MSM and migrants. Their access to healthcare services, including regular testing and prevention programs, varies depending on regional policies and the involvement of NGOs (*p* > 0.05). In contrast, transgender individuals or prisoners are underrepresented (*p* < 0.05).

Recently, NGOs in the Czech Republic have started to make an effort to reach out to transgender people and work with their community to prevent STIs. From a healthcare perspective, prisons are closed communities with their own medical staff. As part of preventive healthcare, emphasis is placed on education about HIV transmission, and testing is also carried out. Employees are also continuously trained on how to behave toward HIV-positive individuals, particularly regarding maintaining confidentiality and the importance of non-discrimination (15). Drug users, however, benefit from well-established harm reduction programs in the Czech Republic, including needle exchange programs and opioid substitution therapy, which have been effective in preventing infections and are relatively well-implemented (*p* > 0.05).

### 3.2 Education

**Figure 2:**
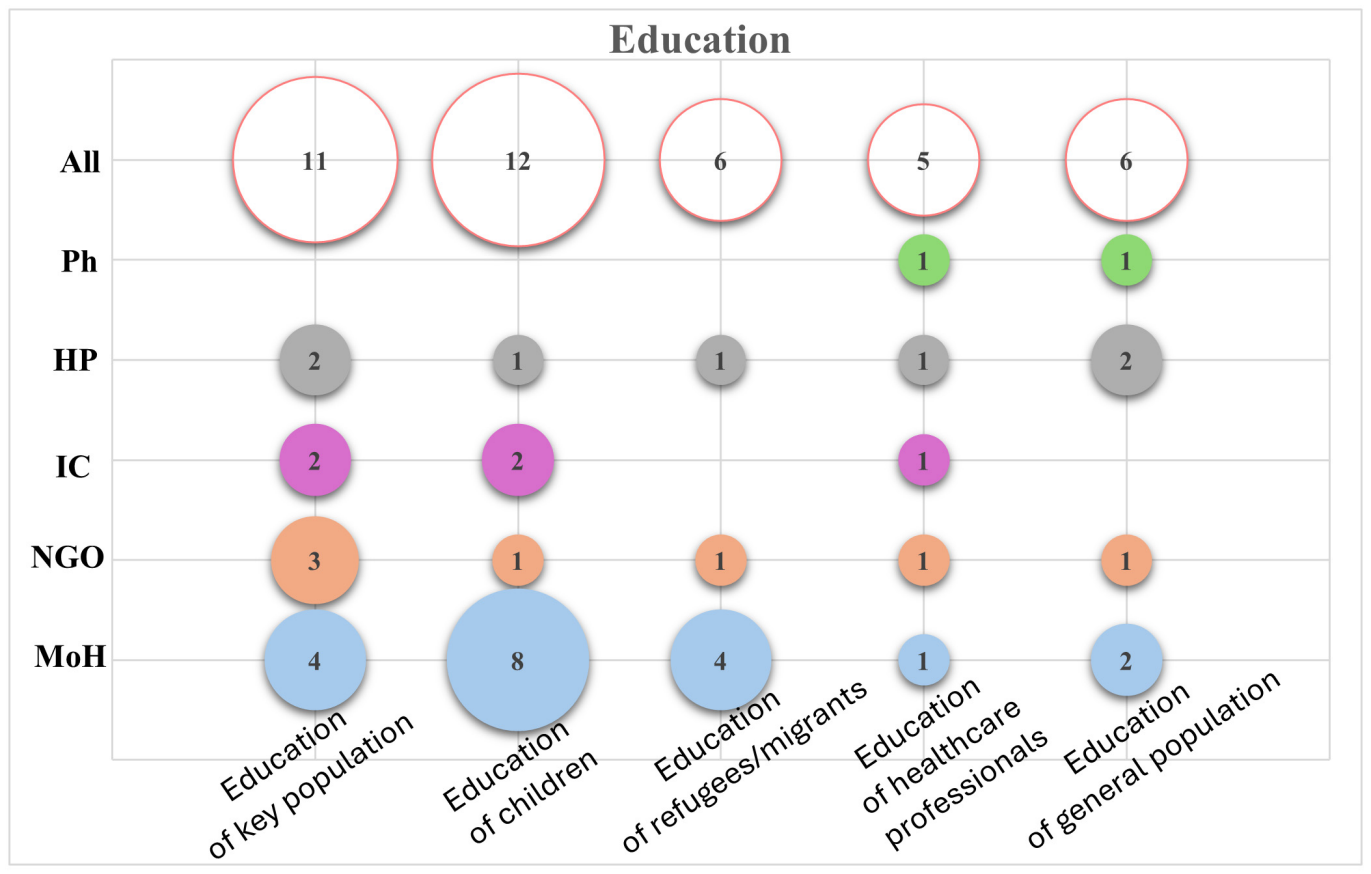
“Education” cluster illustrating stakeholder perspectives on HIV-related educational efforts. The figure presents the frequency of coded references to different target groups for HIV education (e.g., children, healthcare professionals, key populations) across stakeholder categories. Bubble size corresponds to the number of mentions within the qualitative dataset, reflecting the relative emphasis placed on educational activities for each group.

Within the “Education” cluster, we examined the frequency and importance of education for different population groups. We found five codes that represent different groups of people who are or need to be educated about HIV prevention: education of MSM/drug users, children, healthcare professionals, refugees/migrants, and general population. Figure 2 highlights differences in how various groups receive HIV-related education in the Czech Republic.

Stakeholder responses indicate that education efforts are most frequently directed toward children, particularly by the MoH, making this category overrepresented (*p* < 0.05). Refugees and migrants also receive notable education efforts, mainly through NGOs and public health initiatives. Education of key population groups, such as MSM and drug users, is represented to a moderate extent and corresponds to the expected distribution among stakeholders (*p* > 0.05), which indicates that it is receiving adequate attention. According to representatives of all stakeholder groups, the MSM group is well informed about HIV prevention, where they can be tested or where they can get PrEP, which means that this group is clearly educated about risky sexual behavior, prevention, and the impact of prevention tools.

There is a wide range of other preventive tools in the Czech Republic: educational initiatives in schools or at festivals, mobile testing stations, distribution of condoms, and informational materials in nightclubs or at festivals. These preventive initiatives are implemented by non-governmental organizations, the NIPH (National Institute of Public Health) as well as pharmaceutical companies. Education is also provided through websites with information in different languages and contacts to testing centres and PrEP Points, where basic prevention packages (condoms with information) are also available. These educational activities are also aimed at the general public and seek to reach the heterosexual population, which stakeholders perceived as underestimating HIV-related risk in the Czech Republic. Still, educating children remains essential in HIV prevention due to their generally low awareness. However, healthcare professionals are underrepresented in educational efforts, and the general population is also insufficiently targeted (*p* < 0.05). Education of healthcare professionals is also important and should be included in the National HIV Program, as the existence of stigma and discrimination in the healthcare system is a well-known problem.

This suggests that while prevention strategies target high-risk groups, broader public awareness, and professional training are lacking. Some groups receive significantly more attention than others.

### 3.3 Barriers and challenges

**Figure 3:**
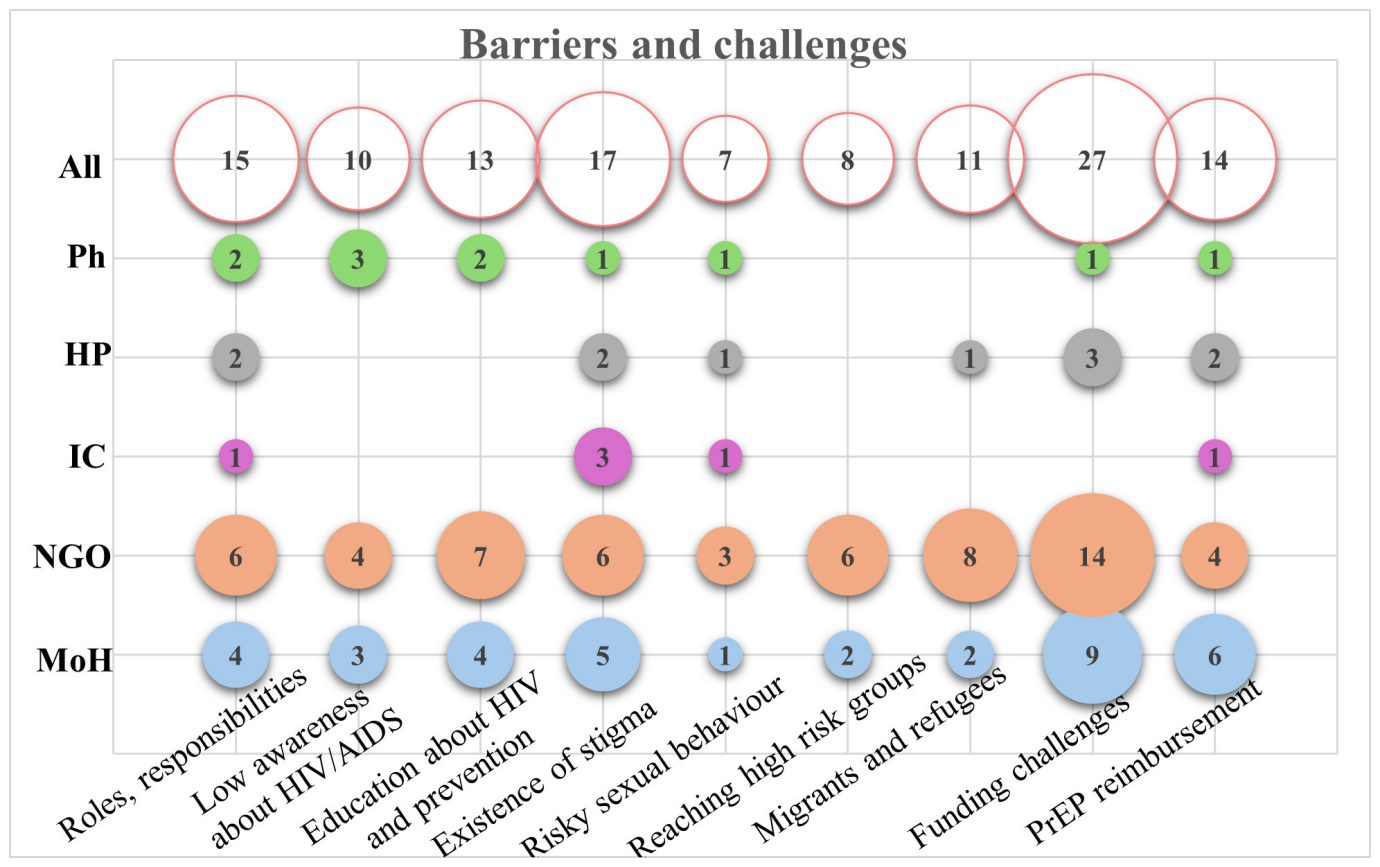
“Barriers and Challenges” cluster identified through qualitative analysis of stakeholder interviews. The figure shows the distribution of coded barriers to HIV prevention (e.g., funding, stigma, awareness, PrEP access) across stakeholder groups. Bubble size indicates the frequency of mentions of each barrier within the dataset, highlighting areas perceived as most critical by stakeholders.

The cluster “Barriers and challenges” includes nine codes: roles and responsibilities, low awareness about HIV/AIDS, education about HIV and prevention, existence of stigma, risky sexual behavior, reaching high risk groups, migrants and refugees, funding challenges, and PrEP reimbursement (Figure 3). We examined the main barriers to HIV prevention and the challenges that should be addressed to make HIV prevention more effective and achieve the 95-95-95 target (29). The most critical challenge, as indicated by the largest representation, is funding challenges, which is overrepresented (*p* < 0.05). PrEP reimbursement also stands out as a major barrier, suggesting that accessibility to PrEP remains an issue in national HIV prevention strategies. Low awareness about HIV/AIDS, and education on HIV prevention are also notable concerns but are more evenly distributed across different stakeholders. The existence of stigma is another key issue, particularly addressed by NGOs and the MoH. Conversely, barriers such as reaching high-risk groups and addressing risky sexual behavior are comparatively underrepresented, suggesting that while they are challenges, they do not appear to be a primary focus of interventions (*p* > 0.05).

Stakeholder research indicates that many stakeholders hold multiple responsibilities in HIV prevention (Table 2). The key areas of responsibility (Table 2) include the National HIV program, education for key populations, strategies for reaching high-risk groups, HIV testing, management of PrEP availability, care for HIV-positive patients, and funding for HIV prevention.

**Table 2:** Roles and responsibilities of stakeholders in HIV prevention.

Stakeholders	Roles and responsibilities
MoH	• National HIV program • Education • Testing
NGOs	• Anonymous testing • PrEP points • Education
HP	• Management of HIV+ patients • Education • Facilitate HIV testing and PrEP prescription
IC	• Reimbursement for HIV-related healthcare services (not yet covered completely)
Ph	• Research on new HIV prevention treatments • Testing • Education • Grants providing

### 3.4 Funding and resources

To identify the resources available for HIV prevention and the challenges related to funding, we created the Funding and Resources cluster. This cluster partially overlaps with some codes in the Barriers and Challenges cluster, highlighting the complexity of HIV prevention efforts. The Funding and Resources cluster includes six codes: National HIV prevention program, targeted funding, long-term planning, harm reduction programs, reimbursement of HIV prevention by insurance companies, and collaboration between stakeholders.

**Figure 4:**
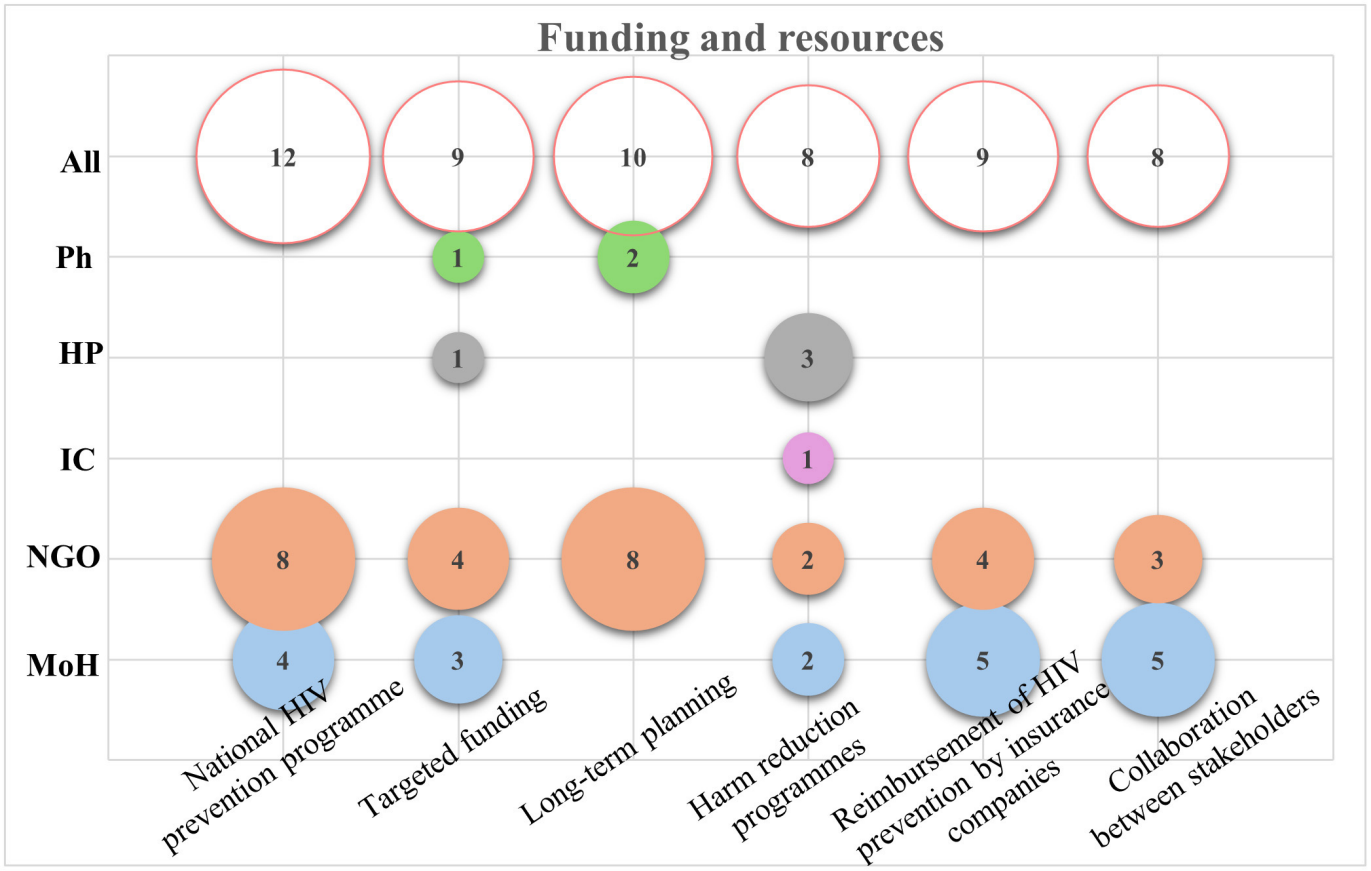
“Funding and resources” cluster representing stakeholder perspectives on financial and structural aspects of HIV prevention. The figure illustrates the frequency of coded references to funding-related themes (e.g., national programs, reimbursement, long-term planning) across stakeholder groups. Bubble size reflects the number of mentions within the coded data, indicating the relative importance assigned to each funding category.

The Funding and resources cluster analysis (Figure 4) highlights key aspects of HIV prevention funding and the involvement of different stakeholders. The most significant funding categories include the National HIV prevention program ruled by NIPH, Long-term planning, and Reimbursement of HIV prevention by insurance companies, as indicated by their high representation. These categories have the largest overall contributions, suggesting they are central concerns in discussions about HIV prevention funding. On the stakeholder level, NGOs and MoH are the most involved actors, contributing to almost every funding category. They play a crucial role in long-term planning, targeted funding, and the National HIV prevention program. MoH is also actively engaged in reimbursement discussions and collaboration efforts.

### 3.5 Expectations

**Figure 5:**
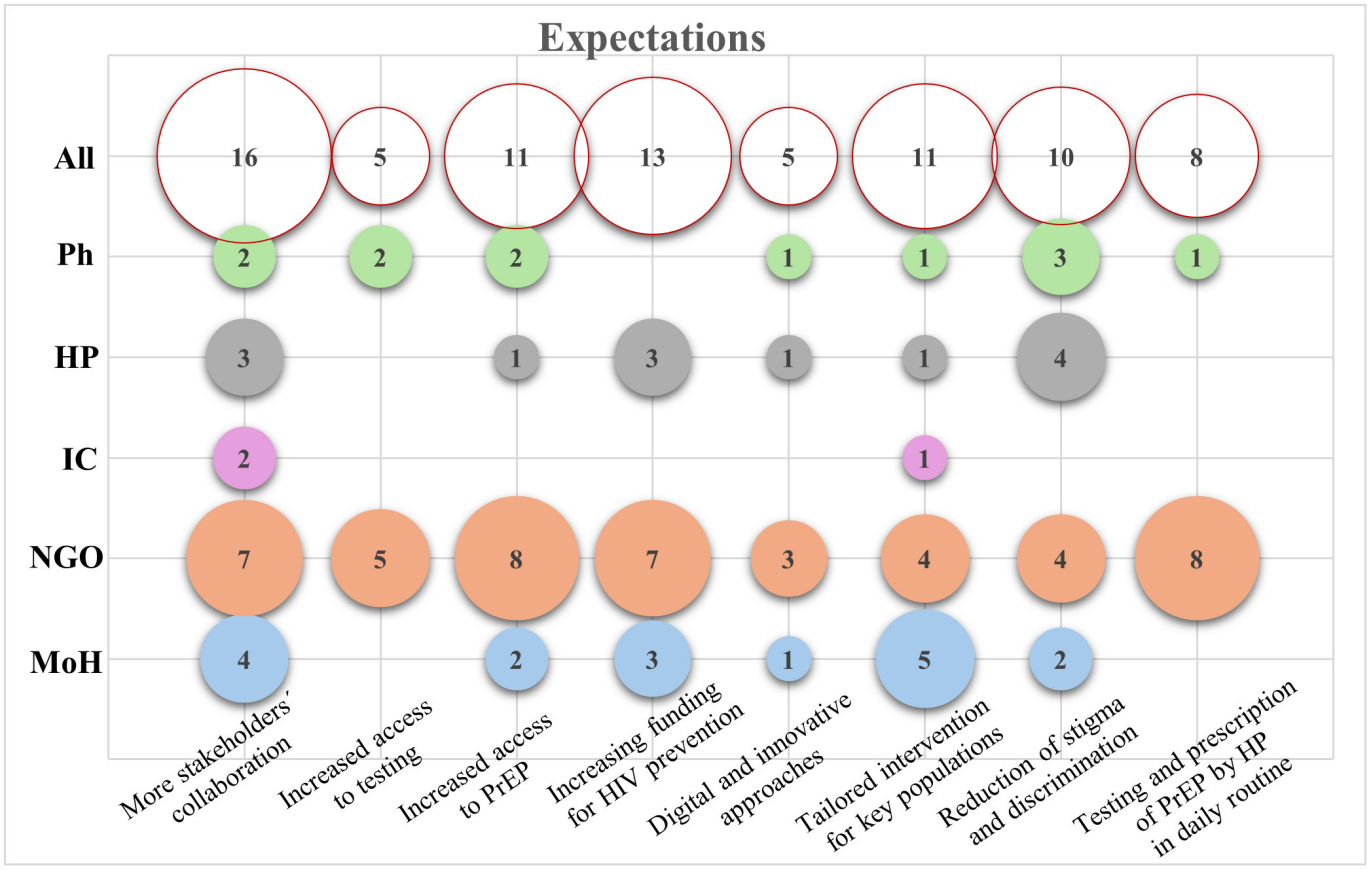
“Expectations” cluster summarizing stakeholder priorities for improving HIV prevention strategies. The figure presents the distribution of coded expectations (e.g., increased funding, stakeholder collaboration, improved access to PrEP) across stakeholder groups. Bubble size represents the frequency of mentions in the qualitative data, indicating the relative importance of each proposed priority.

From an in-depth analysis of discussions with stakeholders, the “Expectation” cluster emerged. This cluster includes eight codes: more stakeholders' collaboration, increased access to testing, increased access to PrEP, increased funding for HIV prevention, digital, and innovative approaches, tailored intervention for key populations and reduction of stigma and discrimination. The “Expectations” chart (Figure 5) shows that the most frequently mentioned priorities include stakeholder collaboration, increased funding, and PrEP availability, indicating strong agreement on these issues.

NGOs underline the need for expanded access to PrEP, additional funding and tailored interventions, while the MoH focuses on stakeholder collaboration and stigma reduction. Healthcare providers stress their role in prescribing PrEP and the need to reduce discrimination, while pharmaceutical companies are mainly concerned with collaboration and digital innovation efforts. Insurance companies (ICs) show limited engagement, suggesting a lack of broader policy engagement.

Importantly, the emphasis on stakeholder collaboration suggests that many actors believe that testing is already well covered and do not see a strong need for further expansion in this area. Greater access to testing was mentioned less frequently, suggesting that stakeholders believe that current efforts are sufficient. While several themes were consistently identified across stakeholder groups, some variation in emphasis and priorities was observed between individual stakeholders.

## 4 Discussion

The stakeholder research on HIV prevention in the Czech Republic has revealed several key areas that require urgent attention, improvement, and strategic collaboration. Through multiple discussions, stakeholders, including government bodies, NGOs, healthcare providers, insurance companies, and the pharmaceutical sector, have expressed their perspectives on challenges, expectations, and necessary advancements. The findings highlight both positive progress and significant gaps that must be addressed to enhance HIV prevention efforts effectively.

While comprehensive HIV prevention includes established tools such as condom and lubricant use, these interventions are already well implemented and accessible in the Czech Republic, as outlined in the Introduction. PEP was not identified as a key priority in stakeholder discussions in the Czech Republic. Therefore, this discussion focuses primarily on gaps and priority areas highlighted by stakeholders, particularly in domains where prevention efforts require further strengthening or development.

At the same time, not all stakeholders emphasized the same priorities, and some differences were observed in how challenges and solutions were perceived across stakeholder groups.

Gokengin et al. (16) conducted a comparative study, where they analyzed the improvement of PrEP usage in 22 European countries. The Czech Republic was the third country with most PrEP offering centres after Ukraine and Poland. The study also examined the barriers to PrEP use or wide use. 59.1% of countries considered the most common reason to be lack of knowledge among people in need, while 50% expressed that the most common reason is that PrEP is not reimbursed. Further barriers identified by countries were low perception of HIV risk (45.5%) and low awareness among healthcare providers (40.9%). 31.8% of countries believed that stigma was a barrier in PrEP usage (16). Our research results align with these findings, as participating stakeholders also identified missing reimbursement and stigmatization as among the most serious obstacles to HIV prevention. Furthermore, our findings regarding “unfavorable funding conditions” (although not directly mentioned in the study) reinforce the financial concerns highlighted by Gökengin et al., confirming that these systemic barriers remain highly relevant.

The future of HIV prevention in the Czech Republic holds great potential, especially with increasing discussions around PrEP reimbursement and expanding prevention efforts to the heterosexual population. As this part of population is becoming more important (in 30 EU/EEA states 46.3% of new HIV cases are heterosexuals) and it rises in Czech Republic as well (from 30.9% to 34% in 2022 resp. 2023) (17, 18). In comparison, heterosexual transmission in 30 European states (EU/EEA, 2022) is 46.3% which is much higher than in Czech Republic and thus prevention must be adjusted to changing situation. While PrEP is currently not covered by health insurance in the Czech Republic, the ongoing dialogue between stakeholders, including the Ministry of Health and NGOs, signals a shift toward a more inclusive and sustainable approach. Studies on cohorts before and after the reimbursement of PrEP show that reimbursement makes PrEP more accessible to a wider group and leads to broader usage (19–21). Ahaus et al. (19) found that the number of sexual encounters in Germany without condoms increased by 16% over a 13-month period in the cohort studied, but no cases of HIV infection were reported.

Our study shows that economic arguments strongly support reimbursement of PrEP, as preventing new infections would save significant healthcare costs. A cost-sharing model, where users contribute partially while insurance covers the rest, is being considered as a realistic step forward. If implemented, this would greatly improve access to PrEP for at-risk individuals who currently cannot afford it. From 2023, in Italy, PrEP is reimbursed by the National Health Service for certain categories of high-risk individuals (22). This decision aligns Italy with the recommendations of other European countries, promoting PrEP as a key preventive option for HIV (23). The WHO European region has national PrEP guidelines, but only 26 countries offer state-funded PrEP, with generics available for purchase in 16 countries and only 13 countries implementing any form of PrEP (23). In the Czech Republic, PrEP is not reimbursed by insurance companies. The gap between PrEP accessibility and expressed need is considerable (12). Even in the UK, a high-income country leading in PrEP prescriptions, there remains an unmet need for access (24). In the WHO European region, expanding PrEP modalities, reducing stigma, and increasing adherence could transform the prevention landscape (25).

Our study uncovered that in Czechia, stigma remains a major problem, especially in the heterosexual population. Unlike in the MSM community, where awareness is higher, many people lack knowledge about HIV. Although the situation in global financing has dramatically changed recently and many achievements could be lost, the belief that HIV is no longer life-threatening and more manageable chronic condition has contributed to a decline in public concern, especially outside Africa region (26). NGOs and the Ministry of Health are addressing this through awareness campaigns and workplace initiatives, but further education and public health messaging are needed to dispel myths and normalize prevention. One such initiative is the #WorkingPositively campaign, supported by major private employers, which promotes inclusive work environments for people living with HIV and other chronic conditions. This project aims to eliminate workplace stigma and foster open dialogue by providing testing access, internal awareness training, and public declarations of inclusivity (27).

Different groups require different prevention strategies. MSM, people who inject drugs, sex workers, and migrants all face unique risks, and prevention efforts should be tailored to their needs. In our study, NGOs highlight that outreach programs, digital campaigns, and peer-led education are effective ways to engage these groups. Pharmaceutical companies suggest using digital tools and targeted online campaigns to spread awareness. Meanwhile, NGOs argue that general practitioners and venereologists should be trained to prescribe PrEP and offer HIV testing, rather than limiting these services to specialist HIV centres. Parczewski et al. also argue that continuous training of healthcare professionals is important to reduce late HIV diagnoses. They highlight the need for strategies based on indicator condition-based testing to achieve the first 95% target in the HIV care continuum (25). According to our research, HIV testing from public insurance in Czechia is available, but access is limited. It is covered only for certain groups, such as pregnant women or blood donors. In Czechia, routine testing is not widely promoted by healthcare system. Public insurance does not support self-request testing. On the other hand NGOs provide anonymous free testing in daily routine, so free testing is accessible only in selected places. By 2023, the Czech Republic had not achieved the UNAIDS 95-95-95 target for awareness of the status of people living with HIV, falling more than 5% short (10). In March 2025, UNAIDS issued recommendations for achieving the HIV prevention target, which remained unchanged at 95%-95%-95% and further, a 90% reduction in new HIV infections between 2010 and 2030 and a continued reduction of 5% per year post 2030 and a 90% reduction in AIDS-related deaths since 2010 (28).

This study has several strengths. It provides an in-depth, multi-stakeholder perspective on HIV prevention in the Czech Republic, incorporating views from key institutional actors across the public sector, healthcare, non-governmental organizations, industry, and payers. The use of qualitative content analysis allowed for a nuanced exploration of stakeholder perspectives, while the combination with quantitative analysis of coded data enabled a structured comparison across stakeholder groups.

However, the study also has limitations. First, participants were recruited using snowball sampling, which may overrepresent well-connected or more visible stakeholders and underrepresent less connected or dissenting voices. Second, the sample size was limited (*n* = 11), and the findings should therefore be interpreted as exploratory rather than statistically representative. Third, patients and members of key affected communities were not directly included, which restricts the perspective to institutional stakeholders. Finally, the use of quantitative comparisons based on coded qualitative data should be interpreted with caution given the small sample size and exploratory design. These findings should be interpreted as exploratory and context-specific, reflecting stakeholder perspectives rather than providing a comprehensive assessment of the national HIV prevention system.

## 5 Conclusion

This study presents the perspectives of selected institutional stakeholders on HIV prevention in the Czech Republic. The findings highlight several key areas identified as priorities, including stronger stakeholder collaboration, improved access to PrEP, more stable funding, and increased public awareness. These findings reflect the views of the interviewed stakeholders and are not representative of all relevant actors. The results should be understood as exploratory insights into current priorities and challenges.

The identified areas may serve as a basis for further research and more targeted policy discussions. Future studies should include a broader range of stakeholders, especially patients and key populations.

## Data Availability

The original contributions presented in the study are included in the article, further inquiries can be directed to the corresponding author.
